# Development and validation of a pentaplex assay for the identification of antibodies against common viral diseases in cattle

**DOI:** 10.1099/acmi.0.000511.v3

**Published:** 2023-10-17

**Authors:** Ana Rodriguez, Rogelio A. Alonso-Morales, Arantzatzu Lassala, Lucia Rangel P, Vianey Ramírez-Andoney, Carlos G. Gutierrez

**Affiliations:** ^1^​ Facultad de Medicina Veterinaria y Zootecnia, Universidad Nacional Autónoma de México, Cd. México, Mexico

**Keywords:** BoHV-1, BRSV, BVDV, EBLV, Multiplex, PI3V

## Abstract

Animal welfare and economic implications of infectious diseases in cattle demand an efficient surveillance as the foundation for control and eradication programmes. Bovine respiratory syncytial virus (BRSV), Parainfluenza virus type 3 (PI3V), Bovine herpes virus-1 (BoHV-1), Bovine viral diarrhoea virus (BVDV), and Enzootic bovine leukosis virus (EBLV) cause common and often underdiagnosed diseases in cattle that are endemic in most countries [1]. A hallmark of individual exposure to a viral pathogen is the presence of antibodies directed towards that virus. The aim of this study was to develop and validate a pentaplex assay to simultaneously detect and quantify antibodies against BRSV, PI3V, BoHV-1, BVDV and EBLV in serum, as an efficient tool to yield epidemiological data. Monoplex assays were initially developed using either complete BRSV or BoHV-1 viral lysates, or recombinant proteins for BVDV, EBLV or PI3V as capture antigens. In addition, 125 serum samples from unvaccinated cattle, which were classified as positive or negative for each of the viruses by commercial ELISA kits, were used for validation. Conditions established for the Luminex monoplex assays were adopted for the pentaplex assay. The accuracy, determined by the area under the ROC curve, was greater than 0.97, and assay diagnostic sensitivities and specificities were over 95 and 90%, respectively, for all antigens. Intra (r) and interassay (R) coefficients of variation were under 10 and 20 %, respectively. Selectivity towards target viruses was shown by binding inhibition assays where unbound viruses reduced fluorescence intensities. Diagnostic agreement for samples analysed simultaneously in the monoplex and multiplex assays was almost perfect. In conclusion, a highly sensitive pentaplex assay was validated for the simultaneous identification of antibodies directed against BVDV, BoHV-1, PI3V, BRSV and EBLV in serum. The developed pentaplex assay complies with performance characteristics established by international guidelines for diagnostic tests and may be used as a tool for the implementation of epidemiological surveillance.

## Data Summary

Data underlying this research can be retrieved using the following link on Figshare: https://doi.org/10.6084/m9.figshare.22341601.v1 [2]. This file has three worksheets with: (1) the mean fluorescent intensities for positive and negative samples used to estimate sensitivities and specificities in the ROC analyses. (2) Mean fluorescent intensities of positive and negative quality controls in the pentaplex assay, and their inter- and intra-assay coefficients of variation. (3) Correspondence analysis of sample status when analysed in either the monoplex or pentaplex formats.

## Introduction

Infectious diseases in cattle have significant animal welfare and economic implications that threaten the sustainability of productive enterprises and affect the quality, quantity and availability of animal products. In addition, lack of specific treatment for viral diseases contribute to the increased use of antibiotics, as they are often associated with secondary microbial infections.

Bovine respiratory syncytial virus (BRSV), Parainfluenza virus type 3 (PI3V), Bovine herpes virus-1 (BoHV-1), Bovine viral diarrhoea virus (BVDV) and Enzootic bovine leukosis virus (EBLV) cause common and often underdiagnosed infectious diseases in cattle that are endemic in most countries according to the WOAH [1]. Bovine respiratory syncytial virus is a paramyxovirus with cytopathic effects, clinical signs of infection are limited to the respiratory system and vary from inapparent to severe [3]. Control is achieved by modified live or inactivated virus vaccines. Morbidity tends to be high (prevalence of antibodies in the US ranges from 60–80 %) and mortality rate spans from none to 20 % [4, 5]. Parainfluenza virus type 3 (PI3V) is also a paramyxovirus that is widely spread in cattle and other ruminant populations, which by itself causes subclinical to mild respiratory signs. However, PI3V infection predisposes the respiratory tract to secondary bacterial pneumonia. Inactivated and modified live vaccines are available for its control [6]. Bovine herpes virus-1 (BoHV-1) infection is associated with multiple disease syndromes in cattle including infectious bovine rhinotracheitis syndrome (IBR), conjunctivitis, pustular vulvovaginitis, balanoposthitis, abortion, encephalomyelitis and mastitis. Adult cattle are the principal reservoir, with high prevalence that indicates a wide distribution [7, 8]. The IBR syndrome, caused by BoHV-1 infection, is rarely fatal in mature cattle unless complicated by secondary bacterial infection of the lung. Despite the low mortality, IBR causes considerable economic losses, and an IBR-free status is required for semen and embryo trade in the European Union [7]. Bovine viral diarrhoea is caused by a pestivirus producing infections that range from subclinical to mucosal disease. It also associates with repeat breeding, abortion, foetal mumification, congenital defects, immunotolerance and persistent infections. Nonetheless, its most important effect is immunosuppression that facilitates the synergistic infection with other pathogens [9]. Enzootic bovine leukosis (EBL) is caused by a retrovirus. An overt cause of economic loss for the producer of EBLV infected cattle is the decommission of carcases affected by lymphomatous tumours. However, more difficult to quantify is the immunosuppression that facilitates secondary infections, which cause suboptimal performance, and a diminished response to vaccination and to natural infections [10, 11]. The economic toll caused from these diseases for bovine producers in North America and Brazil has been calculated in several millions of dollars annually [6, 12].

The implicit animal welfare and economic consequences of infectious disease occurrence, due to decreased productive performance, increased mortality rates, possible reproductive failure, as well as preventive and treatment costs [7, 8, 13]; have led many countries to implement surveillance, control and eradication programmes. Further, as control programmes advance, more rigorous regulations are enacted for commercialization of gametes, live animals and animal products at regional, national and international levels [7] to avoid an increase in prevalence in endemic areas and potential outbreaks in disease-free countries.

Control and eradication programmes require an ongoing intervention through surveillance of pathogen exposure in hosts and susceptible individuals. Initial tests for establishing prevalence of a disease often rely on serological antibody identification, since the antibody registry unequivocally indicates previous or current contact of the population to an infectious agent. Moreover, the development and validation of diagnostic platforms that allow for the detection of antibodies associated to several diseases of interest in parallel (multiplex) in a single sample, potentially provide a detailed snapshot of individual exposure and allow for determination of prevalence rates. Later serological surveillance could confirm the success of vaccination programmes.

Multiplexing technologies have been increasingly used in human health diagnoses, since rapid assessment of several causative pathogen infections and/or differential identification of diseases that have similar symptoms are frequently necessary [14, 15]. In veterinary sciences, multiplexing has predominantly relied on custom assays developed in open platforms that have been mainly tailored to solve particular research demands [14, 16–18], but that have the potential to significantly increase epidemiological resources. The xMAP technology is a microsphere-based assay where beads have unique emission profiles for individual fluorescent wavelength identities that allow users to construct assays with multiple analyte sets. It is a flow-cytometry-like system that allows for each individual microsphere to be queried, and multiple readings are taken per microsphere-set, providing statistical validity and robustness to the data (for details see [19]). The aim of this study was to develop and validate a pentaplex assay, using the Luminex x-MAP platform, to detect and quantify serum antibodies directed against five viral pathogens (BRSV, PI3, IBR, BVD and EBL), as a tool to yield epidemiological data in cattle production units.

## Methods

Viruses were procured from the *American Type Culture Collection* (ATCC) (http://www.atcc.org/): BRSV, Iowa strain (FS1-1), Catalogue No. VR-1485; BVDV, NADL strain, Catalogue No. VR-1422; BoHV-1, Colorado-1 strain, Catalogue No. VR-864; PI3V, SB strain, Catalogue No. VR-739; and EBLV, BL3.1 cells. Catalogue No. CRL–2306, RRID:CVCL 3455. Viruses were grown in Madin−Darby bovine kidney cells (NBL-1; RRID: CVCL_0421) cultured at 37 °C for 6 days in Minimum Essential Medium Eagle with 10 % fetal calf serum, supplemented with penicillin (100 U ml^−1^)/ streptomycin (100 mg ml^−1^)/ amphotericin B (0.25 mg ml^−1^). Infected cell cultures were then subjected to two freeze–thaw cycles and later centrifuged at 4000 ***g*** for 10 min to remove cellular debris. Protein concentrations were subsequently measured in the supernatant with a spectrophotometer at 260 nm. Viral titres were obtained by end-point dilution TCID_50_ using the Reed–Muench method [20].

### Viral antigens

Complete BRSV and BoHV-1 were used as antigens in their respective monoplex assays, as well as in the pentaplex assay. In addition, recombinant antigens were produced for the detection of antibodies against BVDV, PI3V and EBLV in both platforms. Bovine viral diarrhoea virus and PI3V virus RNA was isolated from their respective cell cultures and DNA was purified from EBLV-infected cells. Genes encoding viral antigens were amplified by PCR or RT-PCR. Antigen coding viral gene name and size, as well as primer sequences used for amplification are shown in Table 1 (GenBank access data for the reference genes are: EBLV: AY078387; BVDV: AJ133738; PI3V: AF178655). Primers were designed to include BamH1 sites for cloning and a sequence encoding for six histidines at the 3´ end, for affinity purification. The amplified viral genes were cloned into the baculovirus mobilization vector pFasTBacHa (BacToBac, Invitrogen), following manufacturer recommendations (https://tools.thermofisher.com/content/sfs/manuals/bactobac_topo_exp_system_man.pdf). Briefly, the recombinant pFastTBacHa DNA clones (Thermofisher.com) were used to transform competent DH10BAC cells to generate recombinant bacmids by transposition. The recombinant bacmid DNA was then isolated and employed to transfect SF9 cells to obtain infectious recombinant baculovirus. For recombinant antigen production, the recombinant baculovirus were grown on SF9 insect cells that were cultured for 3 days, and subsequently pelleted by centrifugation at 4000 r.p.m. for 10 min. Pelleted cells were later lysed with 0.1 M Tris at pH 7.5, with 0.15 M NaCl and 1 % Triton X-100, incubated for 30 min on ice, homogenized, and subsequently centrifuged at 4000 r.p.m./10 min. Finally, the supernatant was recovered, and protein content quantified by spectrophotometry at 260 nm.

**Table 1.** Enzootic Bovine Leukosis virus (EBLV), Bovine viral diarrhoea virus (BVDV) and Parainfluenza virus type 3 (PI3V) encoding gene names and sizes, and primer sequences used for amplification

Virus	Gene (size)	Primer sequences
**BVDV**	**E2** (1165pb)	E2DVBF 5’ ATGGATCCGCTGATAACAGGRGYGCAAGG 3’; E2DVBRhis 5’ AT GGATCCTTAGTGGTGGTGGTGGTGGTGTG CTGATAAGACCATGTATGTRACCAG 3’
**PI3V**	**HN** (1719pb)	HNBPI3F 5’ ATGGATCC GATGGAATATTGGAAACACAC 3’; HNBPI3Rhis 5’ ATGGATCCTTAGTGGTGGTGGTGGTGGTG GCTGCAGTTTTTCGGAAC 3’
**EBLV**	**gp51** (1570pb)	BLVF 5’ ATGGATCC GATGCCYAAAG AACGACGGTCCCG 3’; BLVRHis 5’ ATGGATCCTCAGTGGTGGTGGTGGTGGTG GGGCAGGGTCGRAGGTTGATGTAATCG 3’

### Positive and negative quality control sera

One hundred and twenty-five samples (expecting *a 95 % diagnostic sensitivity with 5 % error* [21, 22])*,* from unvaccinated cattle were tested with commercial ELISA monoplex kits (BVDV 99 4400; IBR Ab 99–41459; PI3 P00652-2; BRSV P00651-2; EBLV; P02110-5; Idexx Laboratories, Westbrook, Maine, USA) to establish the negative or positive antibody status for each disease. Later, for validation of monoplex assays in the Luminex x-MAP platform, the ELISA defined positive sera for a single disease, and negative samples for at least the disease being tested, were designated as positive (monoPC) and negative monovalent controls (monoNC), respectively. Seven serum samples resulted positive for all five diseases, these were pooled and used for the validation of the pentaplex assay as a polyvalent positive control (polyC). Since none of the samples tested negative for all five diseases, foetal bovine serum (FBS) was used as the negative control for development and validation of the pentaplex assay.

### Pentaplex assay development and validation

For the development and validation of the pentaplex immunoassay, each individual antigen (recombinant gE/BVD, HN/PI3, gP51/EBLV, and BoHV-1 and BRSV complete viruses) was firstly tested and standardized in monoplex indirect ELISAs to determine the appropriate working concentrations. These were set at 2 µg for recombinant proteins HN (PI3V), gp51 (EBLV) and gE (BVDV). As for BRSV and BoHV-1, 5 µg and 2 µg of complete viruses were respectively used. The concentration of the biotin conjugated secondary antibody (anti-bovine IgG; Jackson Inmunoresearch 101-065-165; RRID: AB_2337297) was set at 1 : 10 000 dilution for all monoplex ELISA assays.

Regarding antibody detection using the xMAP technology from Luminex, five distinctive fluorophore-coded microspheres (1.25×10^6^ microspheres per antigen; BioRad Laboratories, Magplex-C Microspheres, MC10051-01) were covalently bound [23] to the complete viruses or to the recombinant viral antigens, using the carbodiimide method. The antigen–antibody interaction when present was therefore specific and revealed by a second antibody (anti-bovine IgG) labelled with biotin, as an anchor for streptavidin-phycoerythrin (PE) (BioRad Laboratories, 171304501) [23, 24]. The Luminex system allows for the microspheres to be read individually using two laser diodes. The first laser (635 nm) identifies the microsphere code (specific for each virus or viral antigen) and the second laser (523 nm) excites the PE when an antibody is bound to the microsphere-linked antigens [19, 23]. The optimum amount of inclusion of sera in the assay was determined by testing sample dilutions from 1 : 2 to 1 : 32 768. The best antibody binding for all antigens was obtained between 1 : 8 and 1 : 20 dilution. The detection limits of antibody quantification were estimated by serial dilutions of the polyC and reported as the highest dilution that differed from non-specific binding values (NSB) (AOAC 2002).

For monoplex and pentaplex assays in the Luminex platform, 400 antigen-bound microspheres were added per well in a total volume of 50 µl of blocking buffer (PBS 0.01M -Sigma P3813- with 1 % albumin -Sigma 7906- and 0.05 % of sodium azide [NaN_3_] -Sigma 199 931, pH 7.4), for each viral antigen studied. Fifty microlitres of the positive or negative control samples (diluted 1 : 10) were then added to the reaction and plates were incubated for 2 h. Wells were subsequently washed with a PBS solution with 0.05 % tween 20 (Sigma P1379), and 50 µl of biotin-bound anti-bovine IgG were added at 1 : 10 000 dilution and incubated for a further 2 h. After a second wash, antigen–antibody complexes were unveiled by the addition of 50 µl of 1 : 100 PE in blocking buffer. Beads were subsequently rinsed and resuspended in 100 µl wash solution. Mean fluorescent intensity (MFI) for independent antigens was measured and recorded simultaneously by the Luminex platform (Bioplex 100/200, BioRad laboratories). All assays were conducted at 25 °C, with plates protected from light and under constant agitation at 300 r.p.m. throughout.

For validation of the pentaplex assay, antigens were initially evaluated within the Luminex platform as monoplex assays, using the respective monoPC and monoNC to establish cut-off points. Once the monoplex assays were standardized, according to international guidelines (AOAC 2002 [25]), validation of the pentaplex assay was conducted.

Cut-off points (measured in MFI), diagnostic sensitivity and specificity, and positive likelihood ratios (*LR+ >10*) for monoplex and pentaplex assays were determined by the receiver operating characteristics (ROC) curve method (Prism 8 GraphPad Prism – software; RRID:SCR_002798) in triplicate. Cut-off points for each disease were selected for diagnostic sensitivity and specificity values greater than 90 %. The likelihood ratios for the positive results were selected at values above 10 [26].

Pentaplex assay precision was determined by repeatability (r) and reproducibility (R) parameters. For the former, polyC and FBS samples were analysed within the same assay in five replicates. An intra-assay coefficient of variation below 20 % was considered adequate according to international specifications (AOAC 2002 [25]; *Norma Oficial Mexicana NOM-177-SSA1-2013* 2013 [27]; USPC 2019 [28]). For the latter, polyC and FBS were evaluated in quintuplicate on three independent assays. An inter-assay coefficient of variation below 20 % was deemed as adequate (AOAC 2002 [25]; *Norma Oficial Mexicana NOM-177-SSA1-2013* 2013 [27]; USPC 2019 [28]).

Selectivity, shown by antibody binding to its particular antigen [29], was evaluated for BVDV, BoHV-1, and PI3V by binding inhibition monoplex assays. Soluble viral antigens at neat and 1 : 2 dilutions were added to the reaction to inhibit sample antibody binding (monoPC) to the microsphere-linked antigens. In addition, baculovirus expressing haemagglutinin antigen form the H7N3 influenza virus were added as a negative control. These assays were performed in triplicate and the results were expressed as binding percentages.

Agreement on dichotomic results (positive or negative sample status) in monoplex and pentaplex assays was evaluated in simultaneously ran tests. Sixty samples for BoHV-1, BVDV, PI3V and EBLV, as well as 44 samples for BRSV were used, and the agreement between monoplex and pentaplex assays was estimated by corrected Cohen's Kappa coefficient [30, 31].

## Results

**Fig. 1.**
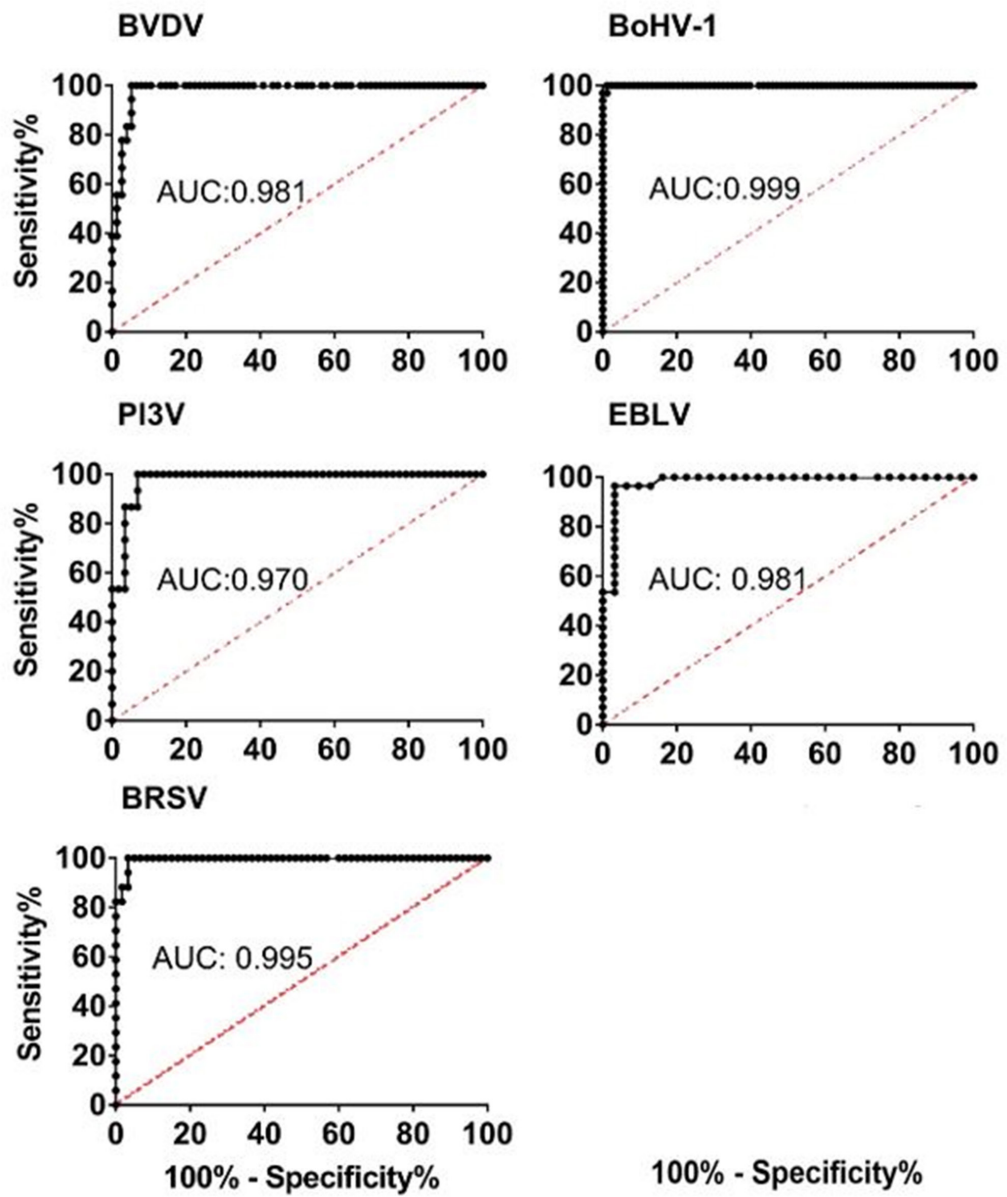
ROC analyses showing diagnostic sensitivity and specificity values at different cut-off points in Luminex monoplex assays to detect serum antibodies directed against Bovine viral diarrhoea virus (BVDV), Bovine Herpes virus-1 (BoHV-1), Parainfluenza virus type 3 (PI3V), Enzootic Bovine Leukosis virus (EBLV) and Bovine respiratory syncytial virus (BRSV).

**Fig. 2.**
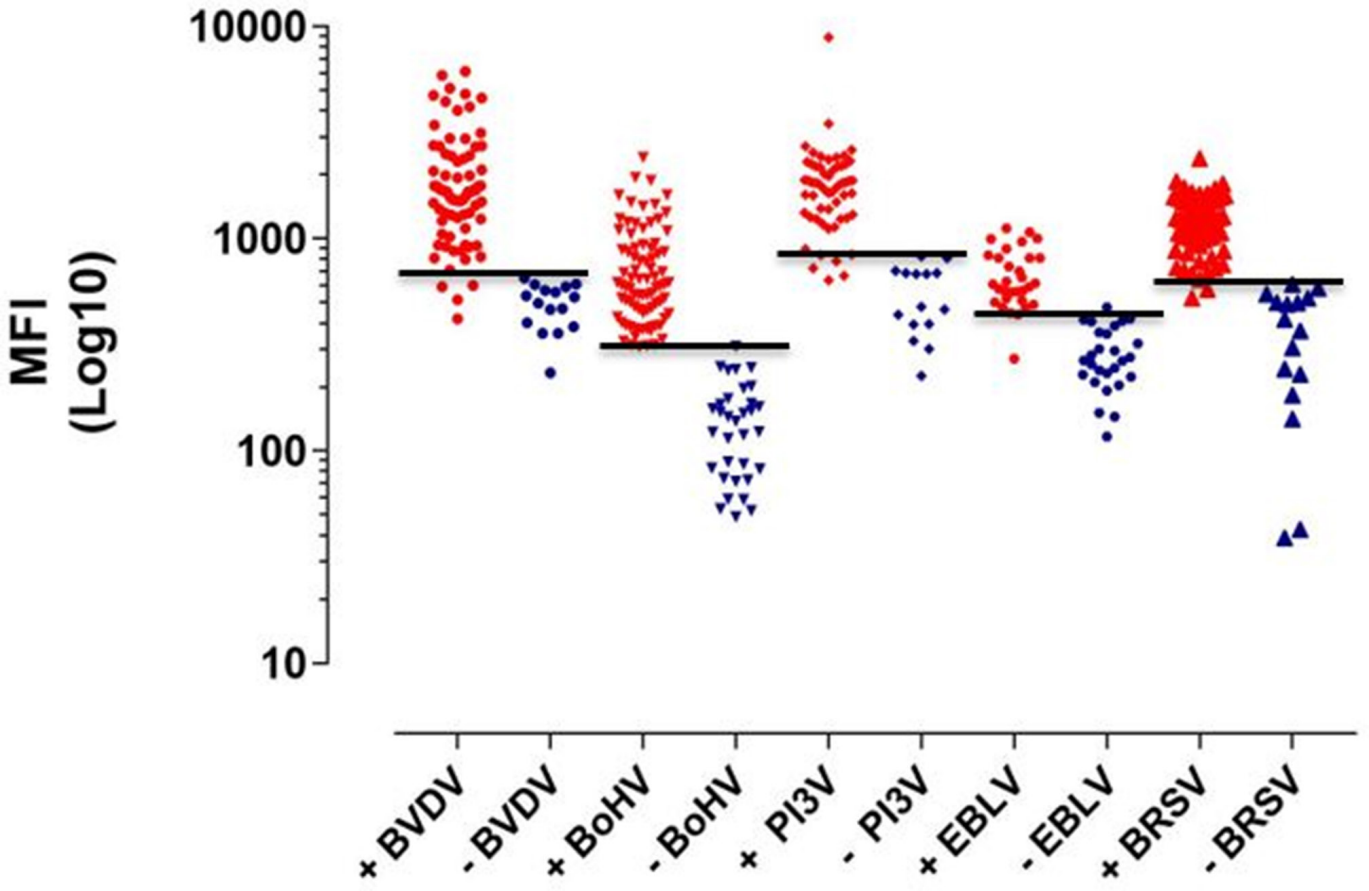
Mean fluorescent intensities for positive (red) and negative (blue) control samples in monoplex assays standardized in the Luminex platform. The horizontal lines show the selected cut-off points for Bovine respiratory syncytial virus (BRSV), Parainfluenza virus type 3 (PI3V), Bovine Herpes virus-1 (BoHV-1), Bovine viral diarrhoea virus (BVDV), and Enzootic Bovine Leukosis virus (EBLV). (+=positive control, -=negative control for each disease tested).

Antibody quantification, which differed from non-specific binding values, was achieved for all antigens tested at sample dilutions between 1 : 2 to 1 : 1024 in both monoplex and pentaplex Luminex assays. To establish optimum true positive rate (sensitivity) and false positive rate (1-specificity) parameters, ROC curves were plotted for mono and pentaplex assays. The area under the curve (AUC) was greater than 0.97 for all antigens (Fig. 1). Mean fluorescent intensities for the positive and negative control samples and optimized cut-off points for viral antibodies are shown in Fig. 2. Diagnostic sensitivity and specificity were over 95 and 90%, respectively, with positive likelihood ratios greater than 10 in all cases (Table 2).

**Table 2.** Performance characteristics of the Luminex Pentaplex assay for Bovine viral diarrhoea virus (BVDV), Bovine Herpes virus-1 (BoHV-1), Parainfluenza virus type 3 (PI3V), Enzootic Bovine Leukosis virus (EBLV), and Bovine respiratory syncytial virus (BRSV), antibody detection. Cut-off points values are shown in mean fluorescent intensity units (MFI). Sensitivity (Se), specificity (Sp), accuracy (AUC), positive (LR+) and negative (LR-) likelihood ratios, and coefficients of variation (CV) for reproducibility (R) and repeatability (r) of at least three assays are shown

Antigen	Cut-off (MFI)	Se (%)	Sp (%)	AUC 95 %	LR+	CV (%)	
						R	r
**gE2 rBVDV**	683.6	100	94.7	0.981 (0.95–1.00)	19.00	11.03	4.28
**BoHV-1**	313.1	100	98.8	0.999 (0.99–1.00)	83.00	10.84	6.39
**HN rPI3V**	833.1	100	93.1	0.970 (0.95–1.00)	14.50	20.00	3.25
**gP51 rEBLV**	430	96.43	96.8	0.981 (0.95–1.00)	29.89	4.93	5.10
**BRSV**	621	100	96.67	0.995 (0.98–1.00)	30.0	9.63	3.86

All assays were deemed as having an excellent precision according to Eurachem [32] and FEUM guidelines, with intra-assay coefficients of variation (r) below 10 %, and inter-assay coefficients of variation (R) lower than 20 % (Table 2).

**Fig. 3.**
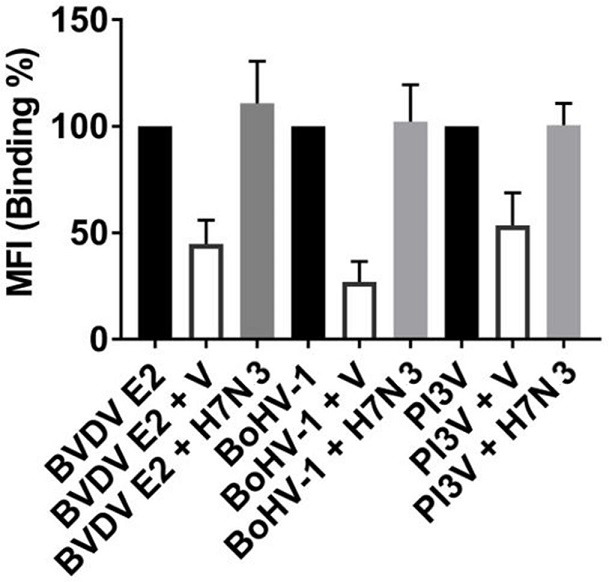
Binding inhibition caused by the addition of unbound viruses to the assay reaction Bovine viral diarrhoea virus -BVDV-, Bovine Herpes virus-1 -BoHV-1-, Parainfluenza virus type 3 -PI3V-. Total binding to the assay antigen (E2 protein of BVDV; complete BoHV-1, or HN for PI3V) in the absence of unbound virus (). The addition of unbound virus caused a reduction in MFI (V;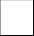). Binding percentage when a baculovirus-H7N3 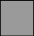; was added to test a potential antibody binding inhibition caused by the baculovirus proteins.

Selectivity towards the viral antigens was shown by binding inhibition assays (Fig. 3) where the addition of unbound BVDV, PI3V and BoHV-1 viruses reduced MFI values. Further, the inclusion of baculovirus expressing influenza H7N3 viral antigens to the assay reaction showed low non-specific non-specificity (<5 %).

To validate the pentaplex assay, established conditions for the monoplex tests were used when all antigens were combined within the same assay (pentaplex platform). The AUCs for the pentaplex assay, which are used to determine sensitivity and specificity parameters, did not differ with those obtained for the monoplex tests (Table 2). Moreover, diagnoses agreement for samples analysed simultaneously in the monoplex and multiplex assays was almost perfect for BoHV-1, EBLV, BRSV and BVDV (>0.81) and substantial for PI3V (0.61–0.80) (Table 3).

**Table 3.** Diagnoses agreement expressed by the corrected Cohen's kappa coefficient, and area under the curve (AUC) for samples analysed simultaneously in monoplex and multiplex Luminex assays for Bovine viral diarrhoea virus (BVDV), Bovine Herpes virus-1 (BoHV-1), Parainfluenza virus type 3 (PI3V), Enzootic Bovine Leukosis virus (EBLV) and Bovine respiratory syncytial virus (BRSV)

Antigen	Monoplex assay AUC	Multiplex assay AUC	Corrected Cohen's kappa coefficient (k´)
**gE2 rBVDV**	0.937	0.960	0.93
**BoHV-1**	0.885	0.852	0.92
**HN rPI3V**	0.846	0.788	0.77
**gP51 rEBLV**	0.832	0.830	0.86
**BRSV**	0.943	0.940	0.96

<0 No agreement; 0.0–0.2 slight agreement; 0.41–0.60 moderate agreement; 0.61–0.80 substantial agreement; 0.81–1.00 almost perfect agreement [30, 31].

## Discussion

A highly sensitive pentaplex assay was validated for the identification of serum antibodies directed against BVDV, BoHV-1, PI3V, BRSV and EBLV. All antigens developed were specific and recognized by antibodies of samples that tested positive by commercial ELISA kits. In addition, a binding inhibition assay proved the selectivity of the antigens to particular viruses or viral antigens. Further, identification of positive and negative samples by monoplex and pentaplex assays had an almost perfect agreement. The detection limit for all diseases was effective up to a 1 : 1024 sample dilution.

For an equivalent number of determinations, multiplex assays have several advantages over monoplex assays. The simultaneous analyses for different antigens, with the reduction in the amount of sample, reagents and time needed for diagnosis are undoubtedly amongst the most important, facilitating epidemiological research and surveillance [24, 33]. However, no guidelines are recognized for validation of multiplex assays, and recommendations published for the standardization of single assays are typically used. Thus, assays need to be firstly validated individually (as monoplex tests) to establish conditions that are subsequently transferred to the multiplex platform [34].

In this study, both monoplex and pentaplex assays for all viral antigens showed diagnostic sensitivity and specificity above 95 and 93%, respectively, with excellent accuracies (ROC AUC >0.96). Receiver-operating characteristics (ROC) analyses calculate sensitivity and specificity parameters over an uninterrupted range of cut-off points (MFI), subsequently plotting the results. The value for the area under the curve (AUC) of such plots indicate the probability of correctly classifying the sample. A trade-off between diagnostic sensitivity and specificity is inevitable, however selected cut-off points for the assay are established when both parameters are above 90 % and the AUC approaches 1, hence complying with the performance guidelines set up by regulatory bodies (AOAC 2002; [25]; *Norma Oficial Mexicana NOM-177-SSA1-2013* 2013 [22]), and efficiently separating between positive and negative samples. Further, given the likelihood ratios observed, the probability that samples classified as positive by the assay come from animals that were indeed exposed to the field virus (since tested sera came from non-vaccinated animals) was at least 10 times greater than those with negative results [35].

Assay selectivity, shown by antibody binding to its particular antigen, was warranted firstly by either using the whole virus (BoHV-1 and BRSV) or by selecting immunodominant and highly conserved antigens present at the viral surface (gp51, HN, and gE2 for EBLV, PI3V, and BVDV, respectively). Secondly, selective binding of serum antibodies to the viral antigens was confirmed by a decreased fluorescent signal in monoplex inhibition tests [22], where soluble antigens were added to the assay reaction. Moreover, the addition of baculovirus-H7N3 to the assay did not cause inhibition of the signal, indicating that baculovirus antigens were not recognized by bovine serum antibodies and thus did not interfere with the outcome. This is relevant as the recombinant antigens used herein were produced in a baculovirus expression system.

Conveyance of performance characteristics from monoplex assays to multiplex assays may be challenging as there is the potential for assay cross-reactivity and interference between analytes [34]. However, since all antigens used for assays in this study belonged to different viral families, the possibility of cross-reactivity was scarce. This assertion was further supported by lack of homology when paired comparisons between the sequences of all antigens were performed (blast data not shown, NCBI GenBank). Hence, serum antibodies directed against an antigen are unlikely to recognize a different antigen non-specifically [29, 36].

High repeatability and reproducibility were observed for all the assays validated (monoplex and pentaplex). The intra-assay (r) and inter-assay (R) coefficients of variation were within the acceptable range (i.e. below 20 %) [32, 36]. Hence, all validated assays had performance characteristics to be qualified for clinical diagnosis.

Taken together, the above evidence indicates that the monoplex assays validated in this work could be successfully merged to a pentaplex platform, without altering performance parameters. Moreover, to effectively assess the diagnostic efficiency of monoplex and pentaplex formats, samples were tested in both platforms simultaneously, finding a substantial agreement for PI3V and an almost perfect agreement for BoHV-1, EBLV, BRSV and BVDV (Table 3) [31]. Thus, an efficient pentaplex assay for simultaneous antibody quantification against five viral cattle diseases with high diagnostic sensitivity, specificity and selectivity was validated for sero-epidemiological studies.

All assays were highly sensitive and able to discriminate the presence of antibodies from the 1 : 2 dilution. Nonetheless, the dilution that characterized the detection limit varied slightly for each antigen, with the 1 : 1024 dilution successfully discriminating positive samples from non-specific binding values for all five antigens tested. Albeit assays could discriminate positive samples in a wide range of dilutions, the MFI was diminished at low dilutions (1 : 8 or below). This counter-intuitive phenomenon (hook effect) occurs when an excess of antibodies impairs their effectiveness to form immune complexes [37]. With high dilution detection limits, a minimal volume of sample is needed for the determination of antibodies without affecting the diagnostic efficiency of the assay. This attribute is particularly useful when analysing samples combined as pools for epidemiological screening, where antibodies of a positive sample could potentially be diluted [37, 38]. A further advantage of a high sample dilution is decreasing potential interference of unrelated sample components in the assay [36], thereby reducing non-specificity. In this regard, non-specificity of the assay is also diminished by the Luminex xMAP platform since antigens are covalently bound to the surface of the fluorescent beads. This contrasts with most other assay types, where antigens are attached to the plate surface by electrostatic adsorption, leaving multiple active sites that could later bind to non-interest molecules, antibodies or the conjugate to create non-specific signals [23, 39]. Moreover, contrasting with assay platforms where the signal is developed and read in the same well where the reaction takes place, bead signal acquisition in the Luminex xMAP assays occur in a capillary where the antigen-linked beads are individually incited by a laser, reducing the possibility of reading non-specific immune complexes formed during incubation [23].

Multiplexing in its various platforms will likely substitute monoplex assays for massive serological surveillance. As with ELISA, xMAP allows for the in-house development of assays, optimizing the antigens of interest and permitting the customization of antigen panels to suit specific needs. The development of an xMAP assay is no more complicated than that of an ELISA that uses the same antigens, in most cases yielding similar or even higher sensitivity. Several multiplex assays have been developed by other research groups to study bovine diseases. Fontana *et al.* [40] created a multi-antigen assay for the serological detection of bovine tuberculosis using recombinant proteins, reporting a lack of interference in the multiplex reaction and similar signal intensity for all antigens used for both the monoplex and multiplex platforms. Others have developed assays for a single pathogen using multiple antigens with satisfactory results (Yun *et al*. 2007 [17]). Anderson *et al*. [16] devised a multiplex assay similar to the one reported herein, where BRSV, BoHV-1, BVDV and PI3V were studied using viral lysates as capture antigens. These authors found that the performance of the multiplex assay was equivalent to that of the monoplex ELISA for two of their antigens (BoHV-1 and PI3V), while it was lower for the other two (BRSV and BVDV). The reason for the later decline in performance is not clear [16]. Nonetheless, the possibility of protein interference or cross-reactivity when multiple viral lysates are used cannot be ruled out. Although a direct comparison between monoplex ELISAs and the pentaplex assay developed in this work was not made, the contrast between antigen performance in the xMap monoplex versus the pentaplex assay was similar for recombinant proteins and viral lysates.

The viruses chosen for the development of the pentaplex assay in this study (BVDV, BoHV-1, EBLV, BRSV and PI3V), have been targeted in eradication/control programmes in Europe and North America due to increasing disease prevalence [10] and the ensuing negative impact in herd health and productivity [7, 41, 42]. Eradication programmes establish prevalence by regions, and encompass preventive medicine programmes and vaccination strategies, as well as the identification, isolation and elimination of positive animals [7, 41, 42]. Nevertheless, most assessments underestimate the potential impact of secondary complications due to immunosuppression and related synergistic effects with other pathogens. Hence, costs of control and eradication programmes should be regarded as an investment, with expenses of diagnostic testing, removal of infected animals, vaccination and monitoring being factored in against reduced losses in the long term. The inception of any control programme is based on accurate knowledge of prevalences. In this endeavour, the development of efficient diagnostic tools is the corner stone for the implementation of epidemiological surveillance and control.
